# Changes in health related quality of life in mothers with inflammatory joint disease from year 2000 to 2020 – a comparative cross-sectional study

**DOI:** 10.3389/fgwh.2024.1458390

**Published:** 2025-01-08

**Authors:** Hege Svean Koksvik, Ingrid Nilssen, Bente Jakobsen, Hilde Bjørngaard, Marianne Wallenius, Kjersti Grønning

**Affiliations:** ^1^ The Norwegian National Network of Pregnancy and Rheumatic Diseases, Department of Rheumatology, St Olavs Hospital Trondheim University Hospital, Trondheim, Norway; ^2^ Department of Neuromedicine and Movement Science, The Norwegian University of Science and Technology, Trondheim, Norway; ^3^ Department of Research, Nord-Trøndelag Hospital Trust, Levanger, Norway; ^4^ Department of Public Health and Nursing, The Norwegian University of Science and Technology, Trondheim, Norway

**Keywords:** motherhood, rheumatic diseases, arthritis, inflammatory joint disease, health related quality of life, women's health, RAND-36

## Abstract

**Objectives:**

More knowledge about health related quality of life (HRQoL) among mothers with inflammatory joint disease (IJD) is needed to understand the complex challenges for this group of patients. The overall aim of this study was to investigate changes in HRQoL among mothers with IJD from year 2000 to year 2020.

**Methods:**

This study had a comparative cross-sectional design with two study groups 20 years apart, year 2000 (*n* = 77) and year 2020 (*n* = 197). Patients were identified from RevNatus, a Norwegian nationwide medical quality register (2020 cohort) and from a national centre for pregnancy and rheumatic disease (2000 cohort). Mothers with the diagnoses of rheumatoid arthritis, juvenile idiopathic arthritis, axial spondyloarthritis and psoriatic arthritis with children aged 0–6 were included. Data on HRQoL were self-reported and assessed by the RAND-36 (SF-36) questionnaire, along with data on educational status, number of children, months since last childbirth and eight questions on experienced motherhood limitations and experienced anxiety and distress for the children. Descriptive statistics were performed using the Mann-Whitney *U*-test, the Pearson chi-squared test and independent samples *t*-test. Multivariable linear regression were used to investigate changes and association between the RAND36 (SF-36) scores and the two study groups and possible confouders.

**Results:**

The 2020 cohort had significantly higher scores on bodily pain (*p* < 0.001), physical function (*p* < 0.001), and role physical (*p* = 0.01) scales compared to the 2000 cohort, indicating better health. There were no significant differences between the two cohorts in the mental health (MH) (*p* = 0.81), vitality (*p* = 0.09), general health (*p* = 0.06), social function (*p* = 0.83), and role emotional (*p* = 0.93) scales. Compared to the calculated norm scores, the 2020 cohort had significantly lower scores on all scales (*p* < 0.01) except on the MH scale (*p* = 0.37).

**Conclusion:**

Mothers with IJD were affected in most dimensions of RAND-36 (SF-36) both in year 2000 and year 2020. The findings emphasize the importance of understanding the intrusiveness of being a mother with IJD despite the improved medical treatment options over the last 20 years.

## 1 Introduction

The impact of inflammatory joint diseases (IJD) like rheumatoid arthritis (RA), juvenile idiopathic arthritis (JIA), axial spondyloarthritis (axSpA) and psoriatic arthritis (PsA) is pervasive and can affect patients' physical and psychological health, social functioning, quality of life and work ability. Untreated, IJD may cause irreversible joint damage and is associated with substantial morbidity and disability compared to the general population. The severity of symptoms and disease activity may fluctuate over time (1–7).

Although there is still no cure for IJD, early diagnosis and aggressive treatment with biological therapies have resulted in substantial major clinical improvements over the past 25 years with significant improvement in symptoms, function, and quality of life. However, many patients still experience that the disease affects many aspects of daily life, even though advances in medical treatment can reduce symptoms (8–15).

The assessment of health related quality of life (HRQoL) in IJD is becoming increasingly common in both research and clinical practice. HRQoL refers to “how health impacts on an individual's ability to function and his or her perceived well-being in physical, mental and social domains of life” (16, 17). One of the most widely used tools for measuring HRQoL is the RAND-36 (equivalent to version 1 of the SF-36 Health Survey) (16–18).

Being a mother is generally considered to be one of the most fulfilling experiences for a woman, but the strain can often be great, having to cope with the problems of the illness and at the same time care for a small child (19). The presence of a painful, disabling chronic disease may have implications for the woman's perceived ability to fulfil a parenting role. Coping with motherhood and having IJD presents not only physical, but also psychological challenges (19). Women with IJD face a range of difficulties with early parenting due to fluctuations in disease activity, treatments for their disease, and difficulties with physical function, pain and fatigue (20–22). The women often stigmatize and blame themselves because they do not live up to their own and society's expectations (23), which may influence their HRQoL. Moreover, the identities of mothers with chronic diseases have been described as dynamic and complex, often experiencing disruption and requiring renegotiation during times of illness relapse or progression (24).

During the last 25 years the literature focus on many of the same challenges in being a mother with IJD with physical limitations; practical and caring issues; social factors; emotional response; hereditary risks; lack of receiving holistic care and appropriate information and education (19, 22, 23, 25–33).

At the Department of Rheumatology, St Olavs Hospital Trondheim University Hospital, a cross-sectional study on HRQoL and experienced limitations in motherhood among mothers with IJD was performed 25 years ago (29). The results showed that the HRQoL scores in the IJD group were lower on most dimensions compared with data from healthy controls. The IJD group also experienced several limitations in motherhood and anxiety/distress about the disease causing extra demands on the children (29).

More integrated care and better information and counselling around early parenting for women with IJD have been recommended (20–22, 33). Previous studies conclude that women with IJD want their motherhood identities and associated preferences to be taken into account in decisions about their healthcare (21, 22, 33). In addition, health professionals need to consider women's multiple and sometimes conflicting identities, and include both their condition and family-associated goals and values in the healthcare (33), in alignment with the core elements of person-centered care. Person-centered care is considered a key component of effective illness management and high-quality care (34, 35) and is by the Word Heath Organization (WHO) emphasized as a core competency of health workers in this endeavor to change and improve the health care system (36). Hence, more knowledge about HRQoL among mothers with IJD is needed to develop individually tailored person-centred care for this group of patients.

The overall aim of this study is to investigate changes in HRQoL among mothers with IJD from year 2000 to year 2020.

**The main objectives:**

• Study differences in HRQoL among two cohorts of mothers with IJD from 2000 to 2020
• Study similarities and differences in HRQoL among mothers with IJD with the norm population
• Study similarities and differences in experienced motherhood limitations and anxiety/distress about the disease among mothers with IJD in 2000 and 2020

## 2 Materials and methods

### 2.1 Study population

This study has a comparative cross-sectional design. The inclusion criteria were women with an IJD diagnosis M05.8, M05.9, M06.0, M07.3+ L40.5, M45, M46.8, M46.9, M08.0 and M08.9, before pregnancy, having one or more children in the age group 0–6.

#### 2.1.1 The 2020 cohort

Patients fulfilling the inclusion criteria were identified from RevNatus, a Norwegian nationwide medical quality register operated by the Norwegian National Network of Pregnancy and Rheumatic Diseases (NKSR). Women with inflammatory rheumatic diseases are prospectively followed in the registry from the time of planning a pregnancy until one year after delivery (37). Patients giving birth in the period 2014–2019 were invited by a postal questionnaire including a written informed consent. The questionnaire included the RAND-36 (SF-36), eight questions about special concerns about being a rheumatic mother, educational status, number of children and months since last childbirth.

Demographic variables and disease-related variables were retrieved from RevNatus.

There were 375 eligible patients with IJD in the register with children aged 0–6 (given birth in the period 2014–2019).

#### 2.1.2 The 2000 cohort

All patients in contact with a national centre for pregnancy and rheumatic disease (located at Department of Rheumatology, St Olavs Hospital Trondheim University Hospital), in the period from January 1996 to May 1999 fulfilling the inclusion criteria were invited to participate (29). There were 119 patients with children aged 0–6 eligible for the study. The eligible patients received a postal invitation letter containing a questionnaire with the RAND-36 (SF-36), eight questions about special concerns about being a rheumatic mother, educational status, number of children and months since last childbirth. The patients consented to participate by returning the questionnaire with no identifiable information.

#### 2.1.3 Reference values/norm data

Norwegian reference values for the RAND-36 (SF-36) were published in 2018 based on a randomly drawn sample representative of the general Norwegian population with respect to age, gender, and place of residence. A norm calculator was conducted by the authors of the Norwegian reference study based on the reference values. The calculator (an excel sheet) is available for free. After entering the characteristics of the study sample, itcalculates expected means based on the characteristics of the study sample (age and gender). This calculator was used for the norm data (38).

### 2.2 Ethics and patient involvement

**The 2000 cohort**: A letter of invitation to eligible patients across Norway was sent with no identifications on the returned questionnaires, making the data unidentifiable. The Research Ethics Committee Health Region IV considered the study, for what approval was granted in March 1999 (REK 42-99).

**The 2020 cohort**: A written informed consent is required before inclusion in RevNatus. The registry was approved by the Regional Committee for Medical and Health Research Ethics (REK) Mid Norway in 2006. The present study also required informed consent and was approved by REK Sout/East Norway in April 2019 (2019/817/REK sor-ost).

Two patient representatives were involved in this study and commented on the design, development and dissemination plans of the project according to EULAR recommendations for the involvement of patient research partners in rheumatology research (39).

### 2.3 Variables

#### 2.3.1 The rand-36 (SF-36)

The RAND-36 (equivalent to version 1 of the SF-36 Health Survey), is freely available and a widely used measure of generic HRQoL (17, 18, 40) and has been translated and validated in Norwegian patients with RA (41). Previous international and Norwegian studies have found RAND-36 (SF36) to be a valid, reliable, and suitable measurement of HRQoL in patients with IJD (41–43) and for young mothers with IJD (29).

The Norwegian version of the RAND-36 (SF36) version 1 was used in both the cohorts and in the reference study (38, 41). RAND-36 (SF-36) consists of 36 items, grouped into eight multi-item scales that measure physical functioning (PF), role limitations due to physical problems (RP), bodily pain (BP), general health (GH), vitality (VT), social functioning (SF), role limitations due to emotional problems (RE) and mental health (MH) (44). Item scores were transformed to 0–100 point scales (0 = worst, 100 = best) using the SF-36 algorithm (44). As per the SF36 algorithm, single imputation was employed meaning that missing values were replaced with the subjects' mean score for the completed items on the same scale if more than 50% of the scale's items were completed (44).

Eight questions on experienced motherhood limitations in caring for the children due to the disease, and experienced anxiety and distress about the disease causing extra demands on the children, were also included in the questionnaire (see Table 1). The questions were derived based on discussions, workshop and a final agreement between five specialist in psychology and a clinical nurse working with mothers with rheumatic diseases. A mean score for the six questions on experienced motherhood and two questions on anxiety/distress was calculated on a scale from 1 (No, none of the time/not limited at all) to 3 (Yes, often/limited a lot).

**Table 1:** Experienced limitations in motherhood and anxiety/distress for the children.

Does the disease limit you in:	2000 cohort mean (SD)	2020 cohort mean (SD)	*p*-value
(a) participating in the child's/children's activities	2.12 (1.12)	1.78 (0.60)	**0** **.** **005** *
(b) practical child care (i.e., changing nappies, dressing, bathing etc.)	1.79 (0.60)	1.51 (0.59)	**<0**.001*
(c) setting limits for your child/children	1.37 (0.56)	1.24 (0.43)	0.08
(d) spending time with the child/children	1.34 (0.53)	1.36 (0.51)	0.75
(e) meeting your own expectations towards the maternal role	1.86 (0.71)	1.81 (0.68)	0.59
(f) meeting others expectations towards your maternal role	1.51 (0.65)	1.40 (0.54)	0.18
Due to your disease, have you been anxious/distressed about:			
(a) your disease causing extra demands on your child/children	1.92 (0.67)	1.79 (0.69)	0.16
(b) your child children being affected by the disease	1.96 (0.66)	1.82 (0.68)	0.13

Independent sample *t*-test.

Bold values, statistical significant.

* Level of significance, *p* < 0.05. SD, standard deviation.

#### 2.3.2 Other variables

Age of respondents at data collection, diagnosis and disease duration were retrieved from patient records (year 2000) or from RevNatus (year 2020) while educational status, number of children and months since last childbirth were self-reported through questionnaire.

### 2.4 Statistical analyses

Characteristics of the study populations are presented as median and inter quartile range or raw numbers and percentages. Descriptive statistics were performed using a non-parametric *t*-test of two samples, The Mann-Whitney *U*-test for non parametric continuous variables and the Pearson chi-squared test for categorical variables and independent samples *t*-test for parametric data. Pearson correlation and multivariable linear regression were used to investigate changes and association between the RAND36 (SF-36) scores and the two study groups, age, number of children, months since last childbirth, educational status, type of disease and disease duration. One sample *t*-test and standardized effect size (Cohens d) was used comparing the RAND-36 (SF-36) of the study population with expected population mean from the norm calculator. The independent samples *t*-test was used to compare the eight questions on “experienced motherhood limitations” and “experienced anxiety and distress” between the two study cohorts. For all statistical tests, a significance threshold of *p* < 0.05 was used. The statistical analyses were performed using IBM SPSS Statistics for Windows, version 29.0.1.

## 3 Results

### 3.1 Response rates

**The 2000 cohort:** Of the 125 questionnaires sent out, 6 were returned because of incorrect address, making a total of 119 eligible for the study. 77 questionnaires were returned, i.e., 65% of eligible subjects responded. No reminder was sent. The characteristics of the non-respondents are unknown.

**The 2020 cohort:** Of the 375 questionnaires sent out, 3 were returned because of incorrect address, making a total of 372 eligible for the study. 197 questionnaires were returned, i.e., 53% of eligible subjects responded. A reminder was sent with the possibility of a web-based response. The characteristics of the non-respondents are unknown.

### 3.2 Descriptive characteristic of the cohorts

Disease distribution, disease duration, age and number of children were similar in the two cohorts. Months since the last childbirth were higher in the 2000 cohort (27.9 months vs. 22.8 months) and there was a difference in educational level with more women in 2020 cohort with higher education (Table 2).

**Table 2:** Characteristics patient groups.

Characteristic	Patients 2000 (*n* = 77)	Patients 2020 (*n* = 197)	*p*-value*
Age (years), median (IQR)	33 (4)	32 (6)	0.09
Number of children (*n*), median (IQR)	2 (1)	2 (1)	0.17
Number of months since last childbirth (months), median (IQR)	22 (33)	18 (21)	**0** **.** **02** *
Educational status *n* (%)			**<0.001** *
Elementary school	13 (17)	5 (3)	
High school	22 (29)	41 (20)	
University college	42 (55)	151 (77)	
Disease *n* (%)			0.93
RA	28 (36.4)	69 (35.0)	
JIA	12 (15.6)	33 (16.8)	
axSpA	27 (35.1)	74 (37.6)	
PsA	10 (13.0)	21 (10.6)	
Disease duration (years), median (IQR)	12 (13)	10 (10)	0.86

Mann-Whitney *U*-test or Chi-Square.

Bold values, statistical significant.

* Level of significance, *p* < 0.05. IQR, interquartile range; RA, rheumatoid arthritis; JIA, juvenile idiopathic arthritis; axSpA, axial spondyloarthritis; PsA, psoriatic arthritis.

### 3.3 Comparison of HRQoL between the cohorts and with norm scores

The 2020 cohort had a significant higher score on the BP andPF scales compared to the 2000 cohort when adjusting for possible confounders. There was no significant difference between the two cohorts in the MH, VT, GH, SF, RP and RE scales (Table 3).

**Table 3:** Linear regression for the SF-36/RAND-36 scales by the two cohorts.

RAND-36 score	2000 cohort, mean (95% CI)	2020 cohort, mean (95% CI)	Univariate analyses		Multivariate analyses^a^	
			Difference between groups, B	*p*-value	Adjusted difference between groups, *β*	*p*-value
Mental health	75.8 (71.8–79.7)	75.3 (73.0–77.5)	0.5	0.81	2.2	0.33
Vitality	37.0 (31.4–41.9)	41.8 (39.9–44.8)	4.7	0.09	1.5	0.62
Bodily pain	44.7 (38.9–50.6)	57.9 (54.4–61.0)	13.2	**<0.001** *	8.7	**0**.01*
General health	48.6 (42.8–53.9)	53.7 (51.0–56.3)	5.4	0.06	2.49	0.40
Social function	71.9 (67.7–79.4)	72.6 (69.0–76.1)	0.7	0.83	2.46	0.49
Physical function	62.6 (58.5–69.5)	77.8 (75.0–80.4)	15.2	**<0**.001*	11.78	**<0**.001*
Role physical	33.4 (25.2–44.3)	49.0 (42.5–55.5)	15.5	**0**.01*	6.21	0.33
Role emotional	78.1 (67.7–86.0)	77.6 (71.3–83.5)	0.6	0.93	7.0	0.24

Higher score indicates better health. Linear regression.

Bold values, statistical significant. Linear regression.

* Level of significance, *p* < 0.05. CI, confidence interval.

^a^ Adjusted for age, number of children, educational status, months since last childbirth, type of disease and disease duration.

We performed univariable linear regression analysis on all eight domains on the possible confounding factors age, number of children, months since last childbirth, number of children, educational level, type of disease and disease duration. For all analysis, the only variable with a linear association in any eight domain was educational level with a linear association with all domains with a *p* value of < 0.05.

In the adjusted analyses the possible confounders; age, number of children, months since last childbirth, number of children, educational level, type of disease and disease duration were in the model. The *p*-value for the score “Role physical” changed from 0.01 to 0.33 after adjusting for confounders.

### 3.4 Comparison of HRQoL between 2020 cohort and norm scores

Compared to the calculated norm score, the 2020 cohort had significantly lower scores on all scales (*p* < 0.01) except the MH scale (*p* = 0.31). The mental health score of 75.3 is of particular interest as high score is described as “feels peaceful, happy and calm all of the time” (Table 4).

**Table 4:** Descriptive statistics for the RAND-36 (SF-36) for 2020 cohort compared to norm data.

RAND- 36 score	2020 cohort, mean (SD)	Norm data, mean	Cohens d (95% CI)	*p*-value
Mental health	75.3 (15.9)	75.7	−0.02 (−0.16 to 0.12)	0.37
Vitality	41.8 (21.1)	52.9	−0.53 (−0.67 to −0.38)	**<0** **.** **001** *
Bodily pain	57.9 (23.4)	78.3	−0.87 (−1.04 to −0.71)	**<0**.001*
General health	53,67 (18.9)	73.6	−1.06 (−1.22 to −0,88)	**<0**.001*
Social function	72.59 (27.9)	84.0	−0.46 (−0.61 to −0.31)	**<0**.001*
Physical function	77.8 (18.9)	93.6	−0.83 (−0.99 to −0.67)	**<0**.001*
Role physical	49.0 (45.8)	84.9	−0.79 (−0.94 to −0.62)	**<0**.001*
Role emotional	77.6 (43.0)	84.8	−0.17 (−0.31 to −0.03)	**0**.009*

One sample *t*-test.

Bold values, statistical significant.

* Level of significance, *p* < 0.05. SD, standard deviation; CI, confidence interval.

### 3.5 Motherhood limitations

Table 1 present the distributions for the items hypothesized to measure motherhood limitations (LIM) and anxiety/distress (ANX) for the children. The scores are summarized making two sum scores; the mean score for LIM on a scale from 1 (No, not limited at all) to 3 (Yes, limited a lot) is 1.7 in the 2000 cohort and 1.5 the 2020 cohort. This might indicate that the mothers are limited to a certain extent. The mean score for ANX on a scale from 1 (No, none of the time) to 3 (Yes, often) is 1.94 in the 2000 cohort and 1.80 in the 2020 cohort indicating that the average of the women are anxious some of the time. There are significant differences in the items “participating in the child's/children's activities” and “looking after the child/children”, with less anxiety/distress in 2020 compared to 2000. In all other items, there are no significant difference between the two cohorts in a 20-year perspective.

## 4 Discussion

This is the first study to examine HRQoL in young mothers with IJD in a 20-year perspective.

Norwegian mothers with IJD were affected in most dimensions of HRQoL in the years 2000 and 2020, compared to Norwegian norm data, indicating that being a mother with IJD still has a pervasive negative effect on many domains of quality of life.

The demographic characteristics of the two cohorts were similar except for the difference in educational level and months since the last childbirth. As expected, more women in 2020 had obtained a higher educational level, as in accordance with the general population in a 20 year perspective (45). We also saw an association with educational level and all the eight scales, with higher education indicating better HRQoL. The difference in months since the last childbirth was 5 months, and the 2020 cohort had the youngest children (23 months vs. 28 months). However, it is unclear whether these 5 months make a clinical difference in perceived mothering. One study has revealed that women generally perceive mothering to be especially demanding during the child's first year of life (46), while another study described the ages from 1 to 2 years to be the most demanding on the mothers (47). Both cohorts had children aged 0–6 years and the mean number of children was not statistical different, assuming the “work load” to be similar between the groups.

### 4.1 Clinical issues

#### 4.1.1 Physical health

The literature states that the impact of IJD on patients is profound and causes considerable morbidity (48–53). Despite continuous improvements in anti-rheumatic pharmacological treatment, people with IJD in general still report substantial disease impact (10, 52, 54, 55).

This study demonstrates that the domains that were most affected among the mothers were the role physical (RP), physical functioning (PF), bodily pain (BP) and general health (GH). These are the most valid measures of physical health problems in RAND-36 (SF-36), and they are also the most valid scales to distinguish between serious and minor medical conditions (56). These domains deal with problems with work or other daily activities as a result of physical health. It is not surprising that RP, PF, GH and BP were most affected if one considers the aspects and consequences of IJD such as pain, stiffness and joint destruction. The 2020 cohort experienced better HRQoL in the BP, PF and RP compared to the 2000 cohort indicating that the physical health has improved during the 20 years, but the mothers still have decreased HRQoL in these domains compared to matched norm data.

We also know that fibromyalgia (FM) is a chronic painful condition frequently associated with IJD and that women with FM have challenges in fulfilling maternal role (57). A systematic review found that concomitant FM is common in chronic inflammatory arthritis, with overall prevalence of FM to be 21% in RA, 13% in ax SpA and 18% in PsA (58). In some cases, IJD inflammation is controlled but the patients still report high levels of pain and associated symptoms (59, 60). Hence, interpreting HRQoL scores may be challenging in IJD with concomitant FM (61).

Problems with daily activities may also include problems with childcare. This may include practical problems of holding the child, especially whilst (breast)-feeding, dressing a baby when fasteners are small and fiddly, and trying to pin down a wriggly baby whilst changing nappies or securing it in a car seat. Our results are in accordance with a qualitative study (62) where the women described RA as a burden. The disease created additional complexity in their daily lives as mothers, due to the physical experiences of fatigue, pain, limited movement, and restrictions to the ability to lift or hold weight (62).

When the child becomes older and more active, there are fears for their safety. Most rheumatic conditions lead to periods of fatigue and general slowness that can make discipline and control more difficult than for fit mothers. Despite improvements in treating joint disease, the extra-articular burden in IJD remains substantial, encompassing multiple comorbidities and psychosocial impairments (8). A recent systematic review shows that more than half of patients with axSpA experience fatigue, with poorer quality of life being associated with more fatigue (63), and fatigue is a frequent symptom in RA (64), in PsA (65) and in JIA (66), with a prevalence up to 70% (67).

#### 4.1.2 Mental health

The mental health (MH) score reported by the 2020 cohort was not significantly different from the norm data, and there were no significant differences between the 2000 and 2020 cohorts either. This is not in concordance with other studies of psychological distress often reported in IJD, where IJD and depression or poor mental health are described as common comorbid pathologies (55, 68–71) that all affect quality of life (72).

A high score of MH in RAND-36 (SF-36) is described as “feels peaceful, happy and calm all of the time”. Interestingly, this study shows that young mothers with IJD, with impairments in many aspects of life, maintain feelings of peacefulness, happiness and calmness. Many factors could account for this, such as the absence of psychiatric disorders, the presence of social supports, the relatively young age and response shift (73, 74) occurring with the management of a chronic disorder. When an individual come to terms with the fact of a long-term illness, adjustments occur that preserve life satisfaction. Thus, chronically ill persons can consider their quality of life as good even when there are severe limitations of their physical ability (73, 75). A previous study also found that personality traits have a considerable influence on how impactful/disrupting patients perceive their disease to be, with decisive consequences on their quality of life (76).

Despite the positive effects associated with motherhood in general, it may also have negative on mothers for mental health. A recent review demonstrates that mothers are at an increased risk for mental distress both during the postpartum period and in the years following postpartum, including an increased risk for depression in the years following childbirth (77). However, this study shows that the great majority of the rheumatic mothers have good mental health.

It has been found that maternal mental health is closely connected to the mother's social support system (78). One can assume that a young woman with a rheumatic disease carefully plans her pregnancy, and makes sure to have a good social support system. Previous studies have also shown that living with a spouse or partner seems to have a positive impact on mental health and life satisfaction (79).

Norway's parental leave policies are generous. Parents have up to 52 weeks of paid parental leave before and after delivery, with at least 15 of these weeks reserved for the father/co-parent. If a new mother is too ill to take care of her infant, the father/co-parent is entitled to take over her part of the leave (80). Universal perinatal programs are in place to safeguard the physical and mental health of the mother and child during pregnancy and after birth. All pregnant women are entitled to free maternity care from a midwife at a maternal and child health (MCH) center or from their general practitioner, and all costs related to delivery and hospital stay are covered. Within the first week postpartum, mothers receive a home visit by a midwife or a public health nurse (81). The MCH centers offer both individual and group-based support for families, addressing difficulties with the child, the parental role and interaction problems. An increasing number of MCH centers have access to psychologists, which has strengthened the implementation of early prevention and treatment efforts for perinatal mental disorders. The maternity and parental well fare system in Norway, might be one of the factors influencing why our finding of good mental health are not in concordance with international literature.

Even though the MH domain was not significantly different from the norm data, it is important to consider that two other domains (SF and RE) that correlate with the summary score for mental health in RAND 36 (SF36) were significantly lower in the patient cohorts than in the norm data. However, these domains also contain elements of physical character (accomplished less, and time spent on social activities) that might be influenced by physical limitations due to the disease.

#### 4.1.3 Experienced limitation and anxiety/distress

Between the two cohorts, the only statistically significant differences were found in experienced limitations in—“participating in the child/children's activities” and—“practical child care (i.e., changing nappies, dressing, bathing etc.)”, with more experienced limitations in 2000 than in 2020. This may reflect the trend in lesser physical limitations due to better medical treatment during the last 20 years.

All other items on experienced limitations and anxiety/distress were not different from 2000 to 2020, with both cohorts experiencing limitation in motherhood and anxiety/distress about the disease affecting their children. We know from other studies that not being able to fulfil their own and others' expectations of their role as a mother can make the women feel dissatisfied (82, 83). The women are concerned about their children and whether the children are experiencing distress, including “health anxiety”, due to witnessing their mother coping with IJD (62). A previous study also reported that women perform self-stigmatization in the form of self-blame and guilt when they cannot accomplish what they want in the mother role because of fatigue, pain, or physical limitations (23), and the women strive to be “good, nurturing mothers”, who are attentive to their children and do what they can to meet their needs (23).

#### 4.1.4 Future recommendations for HRQoL research concerning mothers with IJD

Satisfactory quality of life despite having a rheumatic disease is one of the aims in rheumatology care (2, 84). This implies that explorative research regarding different aspects of quality of life in young mothers with a rheumatic disease is important in the future. In practice, it is less important to know that mothers with IJD have lower mean quality of life scores than healthy controls. The most important question is: what interventions may lead to improved quality of life. Issues under study in rheumatic-related quality of life must therefore have relevance for the care of people with a rheumatic disease.

It would be interesting to explore the basis for the mental health of young mothers with a rheumatic disease. What makes the mental health score of the women in this study higher than in previous studies and research?

Recommendations from EULAR highlight that specific patient educational or self-management programs, at important life and disease course stages, is critical and support patients to make informed choices about how to manage their IJD and facilitate collaborative care (85, 86). It would be interesting to explore if use of person-centered care instruments (34, 35), with targeted patient educational or self-management programs in a rheumatology-led pregnancy clinic (87), would improve the information quality and promote more collaborative decision-making with regard to motherhood and healthcare choices. Since the 2020 cohort have reduced HRQoL in all scores associated with physical health, one can assume that increased improvement in medical care and individualized physical exercises programs could improve these physical aspects of HRQoL and therefore the overall HRqOL.

### 4.2 Strengths and limitations

The main strength of this study is its nationwide patient population and the comparative design, which make it possible to compare two cohorts in a 20 years life span.

A possible limitation is that several different IJD diagnoses are included. One might assume that the HRQoL and disease burden vary across diagnosis. However, Norwegian studies indicate that disease burden in RA, PsA and ax-SpA may be more similar than previously demonstrated (42, 88).

The generic indicator used in this study [RAND-36 (SF36)] does not cover specific aspects required for outcome assessment in patients with RA, JIA, axSpA and PsA. For example, finger function is an important aspect for the assessment of physical health in patients with RA, JIA and PsA, but is not captured by any of the 10 items in the RAND-36 (SF-36) physical functioning scale. Also, we do not have data on clinical disease activity scores nor comorbidity that might influence the HRQoL. Particularly, we know that FM comorbidity impacts overall self-perceived health status (58).

The norm data are matched for age and sex, but not for parity. The total fertility rate in Norway was 1.5 in 2020 (89) and our 2020 cohort had an average of 1.7 children.

## 5 Conclusion

The findings emphasize the importance of understanding the intrusiveness of being a mother with IJD despite the improved medical treatment options over the last 20 years. An understanding of the physical and psychological impact of being a mother with IJD combined with multidisciplinary person-centered care may be a useful goal.

## Data Availability

The datasets presented in this article are not readily available because the data cannot be shared publicly due to the requirements of the involved register holders and the general data protection regulation, to protect the privacy of individuals. Requests to access the datasets should be directed to Hege Koksvik, hege.koksvik@stolav.no.
